# Alleviating the Intestinal Absorption of Rhein in Rhubarb through Herb Compatibility in Tiaowei Chengqi Tang in Caco-2 Cells

**DOI:** 10.1155/2018/7835128

**Published:** 2018-01-30

**Authors:** Ying Peng, Min Fan, Chongsheng Peng, Mengyue Wang, Xiaobo Li

**Affiliations:** School of Pharmacy, Shanghai Jiao Tong University, Shanghai 200240, China

## Abstract

Tiaowei Chengqi Tang (TWCQT) is composed of rhubarb, processed liquorice, and Natrii Sulfas, which is used as a purgative in traditional Chinese medicine (TCM). This study focused on the intestinal absorption of rhein in disassembly of the TWCQT extracts through the Caco-2 cell monolayer model to explicate the possible detoxification mechanism of herb-herb compatibility in TWCQT. The results showed that the intestinal absorption of rhein occurred through active diffusion, and rhein might be composed of breast cancer resistance protein (BCRP) substrates. The extract of processed liquorice increased the exclusion rate and reduced intracellular uptake of rhein. The consistent results observed in TWCQT further implied that processed liquorice in TWCQT could suppress the absorption of rhein across the Caco-2 cell monolayer. It has therefore been concluded that the active ingredients of processed liquorice may play a critical role in reducing the intestinal absorption of rhein to alleviate the toxicity of rhubarb in TWCQT. Because of BCRP's involvement in rhein transport, we conjectured that some components in processed liquorice could inhibit the transport of rhein, possibly by mediating BCRP. These results would provide new insight into this ancient drug combination in toxicity reduction and clinical use.

## 1. Introduction

Tiaowei Chengqi Tang (TWCQT), recorded in* Shang Han Lun* and consisted of three herbs, rhubarb* (Radix et Rhizoma Rhei, *Polygonaceae), processed liquorice* (Glycyrrhizae Radix et Rhizoma Praeparata Cum Melle)*, and Natrii Sulfas, with a ratio of 4 : 2 : 3, has been traditionally used as a purgative to relieve constipation and to clear internal heat in the stomach and intestine [1]. Modern pharmacological research has proven that TWCQT possesses properties of detoxification, antipyresis, and adjustment of the gastrointestinal tract [2].

Multiherb prescription is recognized as a unique pattern of traditional Chinese medicine (TCM) clinical application, in which herbs work together to exert therapeutic actions or modulate the pharmacological and toxicological effects [1]. According to the compatibility principle of TCM, rhubarb is the principal drug in TWCQT [3]. Rhubarb is officially listed in the Chinese, European, and Japanese Pharmacopoeia, commonly used in TCM prescriptions, as well as in self-medication for constipation in many countries [4]. Anthraquinones are reported to be the major active components present in rhubarb with wide pharmacological effects and some toxicity concerns [5–7]. Among these, rhein is a key compound to understand rhubarb's toxicity and effectiveness. Rhein was the most absorbable anthraquinone derivative into blood circulation after oral administration of rhubarb extract in rats [8]; meanwhile, rhein was identified as one of the toxic anthraquinones [9, 10]. These evidences suggested that rhein had the good strong protein binding affinity and bioavailability to cause toxic manifestations in rhubarb.

It has been reported that the concentration of rhein in plasma is reduced following oral administration of TWCQT compared to rhubarb extract, indicating the alleviation of rhubarb toxicity by TWCQT [11]. Moreover, the concentration of rhein was decreased when liquorice was compatible with rhubarb in rats [12]. Liquorice could inhibit the intestinal motility in rats [13] and induce CYP3A enzyme [14], which thereby could relieve the abdominal pain caused by rhubarb and accelerate the metabolism of anthraquinones. While the combination therapies of TWCQT have been validated and show potential clinical benefits, herb-herb interactions in prescriptions have not yet been fully clarified.

In the present study, we employed the Caco-2 cell monolayer model to investigate the transport difference of rhein alone and rhein in different decoctions, including rhubarb extract (RE), rhein with processed liquorice extract (rhein + PE), rhubarb with processed liquorice extract (RPE), and TWCQT and their mechanisms. The aim of this study was to clarify whether the intestinal transport of rhein is influenced by other components in rhubarb, the effects of prepared liquorice, and TWCQT on the intestinal absorption of rhein.

## 2. Materials and Methods

### 2.1. Herbs

Rhubarb, Natrii Sulfas, and processed liquorice were purchased from Leiyunshang Pharmacy of Leiyunshang Pharmaceutical Co., Ltd. (Shanghai, China). All herbs were authenticated by Professor M. Y. Wang, an experienced botanist specializing in medicinal herbs. Voucher specimen (20151102-a, b, c) has been deposited at herbarium of School of Pharmacy, Shanghai Jiao Tong University, Shanghai, China.

The crude slices of rhubarb (8 g) were extracted twice with 8 times volume boiling water for 30 min each time. The filtrate was concentrated to prepare the stock solution of 1 mg/mL for rhubarb. Prepared liquorice (4 g) and TWCQT (rhubarb 8 g, Natrii Sulfas 6 g, and processed liquorice 4 g) were processed by the same approach to obtain the corresponding stock solution. The amounts of rhein in the 1 mg/ml RE, RPE, and TWCQT were determined by ultra-high-performance liquid chromatography-quadrupole time-of-flight-high-resolution mass spectrometry-mass spectrometry (UPLC-QTOF-HRMS-MS). All stock solutions were further diluted with D-Hanks to obtain a series of solutions with required concentrations.

### 2.2. Reagents and Chemicals

The human colon adenocarcinoma cell line, Caco-2, was purchased from the Cell Bank of the Academy of Science (Shanghai, China). Rhein was purchased from Man Site Biotechnology Co., Ltd. (Sichuang, China). Dulbecco's Modified Eagle's Medium (DMEM, high-glucose 4.5 g/l), nonessential amino acids (NEAA), and penicillin and streptomycin solution (10,000 U/ml penicillin and 10,000 *μ*g/ml streptomycin) were purchased from Genome Bio-Medical Technology Co., Ltd. (Hangzhou, China). Fetal bovine serum (FBS) was purchased from Hangzhou Sijiqing Co., Ltd. (Hangzhou, China). Trypsin-ethylenediaminetetraacetic acid (EDTA) solution (0.25%) was obtained from Beijing Dingguo Changsheng Biotechnology Co., Ltd. (Beijing, China). Verapamil, MK-571, and Ko143 were purchased from Sigma-Aldrich (St. Louis, MO, USA). Millicell cell culture inserts were obtained from Millipore (USA). Twenty-four-well cell culture clusters were obtained from Costar (Corning Incorporated, USA).

### 2.3. Caco-2 Cell Culture and Cytotoxicity Assay

Caco-2 cells were maintained in DMEM containing 10% FBS (inactivation at 56°C for 30 min), 1% NEAA, and 1% penicillin and streptomycin solution in a humidified atmosphere with 5% CO_2_ at 37°C. The medium was replaced every 2 days, and the cells were passaged at 80 to 90% confluence using trypsin/phosphate-buffered saline (PBS, pH 7.4). The cytotoxicity of rhein, RE, PE, RPE, and TWCQT to Caco-2 cells was evaluated by MTT assay. Briefly, 200 *μ*l of Caco-2 cells was seeded in a 96-well plate (Coring, NY, USA) at a density of 2 × 10^4^ cells/ml. Following incubation for 24 h, different concentrations of test solution were added. Thereafter, 20 *μ*l of 5 mg/mL MTT was added, the medium was removed after 4 h, and the remaining formazan crystals were solubilized with 150 *μ*l of dimethyl sulfoxide (DMSO). The absorbance was measured at 490 nm on a microplate reader (Thermo Scientific, Tokyo, Japan). Cells incubated without the test samples were used as controls. In each MTT assay, every sample was tested in 6 replicates.

### 2.4. Transport of Analytes across the Caco-2 Monolayer

For transport experiments, the cells were seeded at a density of 1 × 10^5^ cells/ml on polycarbonate membranes of cell culture inserts (6.5 mm membrane diameter, 0.4 *μ*m pore size, and 0.33 cm^2^ surface area) and placed in 12-well cell culture clusters. The transepithelial electrical resistance (TEER) was assessed using a Millicell ERS-2 (Millipore, USA) to reflect the tightness of intercellular junctions. The Caco-2 cell monolayers were used for transport experiments on day 21 of postseeding with TEER values >500 Ω cm^2^.

The transports of rhein alone, rhein in RE, PE, RPE, and TWCQT across Caco-2 monolayers were investigated. Briefly, the cell monolayers were washed 3 times with D-Hanks. After each wash, the plates were incubated in fresh D-Hanks for 30 min at 37°C. The transport experiments were conducted by adding the test solution to either the apical (A, 0.5 ml) or basolateral side (B, 1.5 ml), while the receiving chamber contained the corresponding volume of prewarmed drug-free D-Hanks. Every experiment was repeated 3 times, and the plates were incubated in an orbital shaker at 37°C. Six sequential samples (50 *μ*l) were taken at different times (30, 60, 90, 120, and 150 min) from both sides of the cell monolayer. The same volume of D-Hanks was immediately added to replace the samples obtained. Transport experiments of rhein were also performed in the presence of efflux transporter inhibitors (i.e., verapamil, Ko143, and MK-571). All inhibitors were loaded onto the A side of the monolayers. Verapamil is a P-glycoprotein (P-gp) inhibitor, Ko143 is a breast cancer resistance protein (BCRP) inhibitor, and MK-571 is a multidrug resistance protein 2 (MRP2) inhibitor. The concentrations of all the samples were analyzed by UPLC-QTOF-HRMS-MS.

### 2.5. Cellular Uptake of Analytes in Cultured Caco-2 Cell

Cell monolayers were prepared as described for the transport studies. Test solution were loaded onto the A side of the cell monolayers over 2 h at 37°C. Cells attached to the polycarbonate membranes were cut off from the inserts, immersed in 1 ml blank D-Hanks, and sonicated for 15 min. The mixture was centrifuged at 10,000 ×g for 5 min. The concentrations of all the samples were analyzed by UPLC-QTOF-HRMS-MS.

### 2.6. UPLC-QTOF-HRMS-MS Analysis

Analyte measurement was performed on a Waters ACQUITY UPLC system (Waters Corp., Milford, MA, USA). Chromatography was carried out on an ACQUITY UPLC BEH C18 column (100 mm × 2.1 mm i.d, 1.7 *μ*m, Waters Corp., USA). The mobile phase consisted of 0.1% formic acid (A) and acetonitrile (B), using a gradient elution of 13% B at 0–8 min, 27.5% B at 8–14 min, 37.5% B at 14–25 min, and 100% B held for 2 min. The gradient was recycled back to 13% in 2.5 min for the next run. The flow rate was 0.6 ml/min. The detection wavelengths were set at 254, 270, and 340 nm. The injunction volume was 3 *μ*l. The temperature of the column oven was set to 35°C.

Mass spectrometry was carried out using a Waters SYNAPT mass spectrometer (Waters Corp., Milford, MA, USA). Ionization was performed in the negative electrospray ionization (ESI) mode. The MS parameters were as follows: capillary voltage, 2.8 kV; cone voltage, 35 V; source temperature, 115°C; desolvation temperature, 350°C; gas flows of cone and desolvation, 50 and 700 l/h, respectively. For accurate mass measurement, leucine enkephalin was used as the lock mass. The MSE experiment in two scan functions was carried out as follows: function 1 (low energy), *m*/*z* 50–1000, 0.25 s per scan time, 0.02 s interscan delay, and 4 eV collision energy; function 2 (high energy), *m*/*z* 50–1000, 0.25 s per scan time, 0.02 s interscan delay, and 4 eV collision energy ramp of 55–70 eV.

### 2.7. Data Analysis

Data in the present study were presented as mean ± standard deviation (SD). Significant analysis was performed using Statistical Package for Social Sciences (SPSS) version 16.0 (SPSS Inc., Chicago, IL, USA). Statistical significance was determined by one-way ANOVA with Dunnett's test and differences were considered significant when* p* < 0.05. In the Caco-2 cell model, the rate of transport was obtained from the amount transported versus time curve using linear regression. The apparent permeability (*P*_app_) used as an expression of the absorption rate constant was calculated as follows: *P*_app_ = (*dQ*/*dt*)/(*A* × *C*_0_), where *dQ*/*dt* is the rate at which the compound appears in the receiver chamber (mg/s), *A* is the surface area of the filter membrane (1 cm^2^), and *C*_0_ is the initial concentration in the donor chamber (mg/mL). Efflux ratio (ER) was calculated from the following equation: ER = *P*_app_(B to A)/*P*_app_(A to B), where *P*_app_(B to A) is the *P*_app_ value measured in the B to A direction and *P*_app_(A to B) is the *P*_app_ value measured in the A to B direction.

## 3. Result

### 3.1. Quantification of Rhein in RE, RPE, and TWCQT by UPLC-QTOF-HRMS-MS

**Figure 1:**
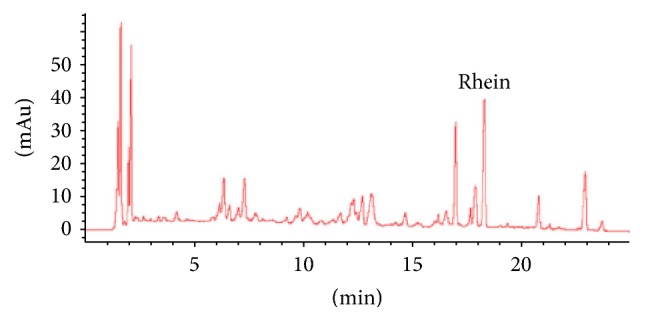
Typical UPLC-QTOF-HRMS/MS chromatograms of TWCQT.

The amounts of rhein in the 1 mg/ml RE, RPE, and TWCQT were determined by UPLC-QTOF-HRMS-MS (Figure 1). The results showed that the amounts of rhein in RPE (1 mg/ml) and TWCQT (1 mg/ml) were 27.7 *μ*g/ml and 27.3 *μ*g/ml, respectively; however, in RE (1 mg/ml) this amount was 8.61 *μ*g/ml. This may be explained by processed liquorice's ability to improve the extraction rate of rhein in rhubarb during codecoction. Therefore, the concentrations of rhein in RE (8.61 *μ*g/ml) and TWCQT (27.3 *μ*g/ml) served as the references for uptake and transport experiments in cultured Caco-2 cells.

### 3.2. Cytotoxicity of Rhein, RE, PE, RPE, and TWCQT on Caco-2 Cell

To ensure cell viability during the permeability experiments, viability of cells was directly measured using an MTT test to evaluate the cytotoxicity of rhein, RE, PE, RPE, and TWCQT toward Caco-2 cells prior to transport experiments. Generally, a higher cell viability of more than 90% indicated that the compounds at the concentrations were nontoxic to the cells. The results showed that rhein at or below the concentration of 200 *μ*g/mL was nontoxic to the Caco-2 cells after 48 h exposure (IC50 3.49 ± 0.96 mg/ml). RE, PE, RPE, and TWCQT at concentrations up to 4 mg/mL showed no toxic effects to Caco-2 cells (IC50 19.71 ± 0.96, 19.88 ± 1.34, 23.70 ± 2.52, and 21.87 ± 0.53 mg/ml, resp.).

### 3.3. Transport of Rhein through Caco-2 Cell Monolayers and Effects of Inhibitors

**Figure 2:**
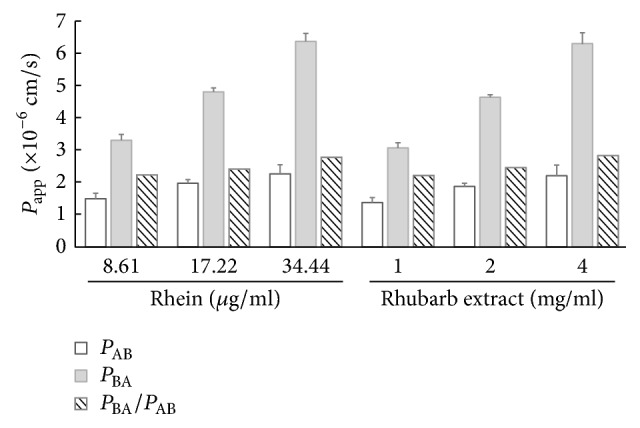
Effects of rhubarb extract on transport of rhein across the Caco-2 cell monolayer. The amounts of rhein in 1, 2, and 4 mg/ml RE were 8.61, 17.22, and 34.44 *μ*g/ml, respectively. *P*_AB_: the apical to basolateral side; *P*_BA_: the basolateral to apical side.

The transports of 8.61, 17.22, 34.44, 27.3, 54.6, and 109.2 *μ*g/ml rhein across a Caco-2 cell monolayer from sides A to B and B to A were investigated (Figure 2 and Table 1). The *P*_app_ values of rhein from B to A were significantly (*p* < 0.05) higher than those from A to B. The ERs of rhein at different concentrations were all more than 1.5, which indicated that rhein could be absorbed across intestinal epithelial cells in active absorption patterns, and its transport process might be mediated by some transporters.

**Figure 3:**
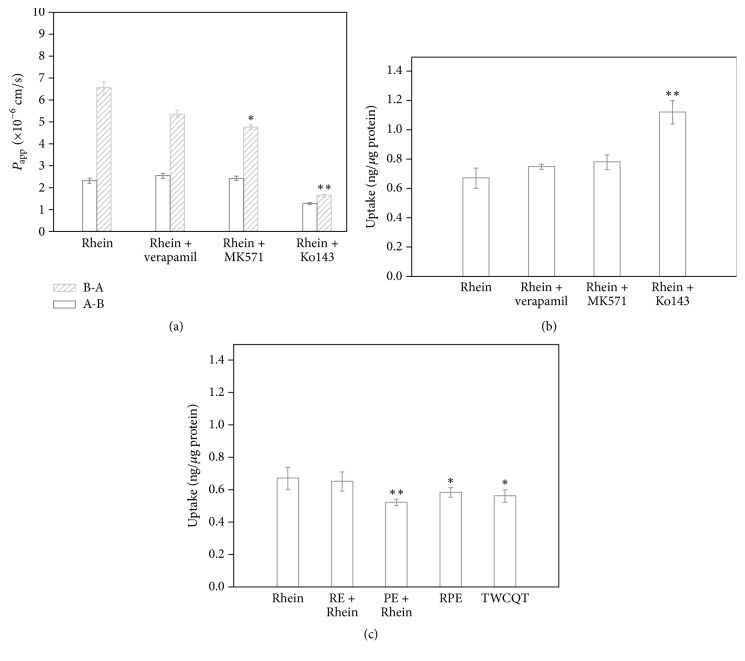
Effects of inhibitors on permeability (a) and uptake (b) of rhein and effects of RE, PE, RPE, and TWCQT on uptake (c) of rhein by Caco-2 cells. B-A: the basolateral to apical side; A-B: the apical to basolateral side. Verapamil: 50 *μ*mol/ml; MK-571: 50 *μ*mol/ml; Ko143: 10 *μ*mol/ml; rhein: 27.3 *μ*g/ml rhein; RE + rhein: 1 mg/ml rhubarb extract + 18.69 *μ*g/ml rhein; PE + rhein: 1 mg/ml prepared licorice extract + 27.3 *μ*g/ml rhein; RPE: 1 mg/ml rhubarb + prepared liquorice extract; TWCQT: 1 mg/ml Tiaowei Chengqi Tang. Data are represented by the mean ± SD from three replicates. ^*∗*^*p* < 0.05; ^*∗∗*^*p* < 0.01 compared with 27.3 *μ*g/ml rhein.

The effects of efflux transporters on rhein are shown in Figure 3(a). Verapamil (50 *μ*mol/ml) reduced the amounts of rhein (27.3 *μ*g/ml) transported from B to A, and induction of ER decreased to 2.12, but with no significant influence on the *P*_app_ values of rhein from A to B or B to A. MK-571 (50 *μ*mol/ml) showed significant influences on the *P*_app_ values of rhein from B to A (*p* < 0.05) and resulted in decreased ER (1.98). The above results suggest that MRP2 might mediate the transport of rhein. After treatment of 10 *μ*mol/ml of Ko143, the amounts of rhein transported from B to A significantly decreased (*p* < 0.01) and caused the decrease of ER (1.27), indicating that BCRP may be involved in the transport of rhein and perform an important role.

### 3.4. Transport of Rhein in RE, PE, RPE, and TWCQT through Caco-2 Cell Monolayers

The transport flux of rhein across the Caco-2 in RE, PE, RPE, and TWCQT is shown in Figure 2 and Table 1. No significant differences were observed in the *P*_app_ values for rhein in 1, 2, and 4 mg/ml RE compared with corresponding concentrations of rhein alone, suggesting that other components in RE did not alter the membrane permeability of rhein in Caco-2 cells (Figure 2). However, the A to B flux and the B to A efflux of 27.3, 54.6, and 109.2 *μ*g/ml rhein in Caco-2 cells were significantly inhibited (*p* < 0.05), causing an increase in ER (3.20–3.53, approximately 1.3-fold compared to rhein alone) in 1, 2, and 4 mg/ml of PE (Table 1). The *P*_app_ values of rhein determined in RPE and TWCQT were consistent with those of PE, showing similar reduction in both directions with ER values of 3.16 to 3.66. These results indicated that processed liquorice in TWCQT might inhibit the absorption of rhein in Caco-2 cells (Table 1). Generally, transport flux of a compound with ER values more than 1.5 is considered as an active efflux. Results obtained from the study of transport of rhein in PE, RPE, and TWCQT were all greater than 3. Therefore, the mechanism of permeation for rhein in PE, RPE, and TWCQT in the translocation across Caco-2 cell monolayers was supposed to involve active efflux, and some components in processed liquorice, which may be the substrate of MRP or BCRP, would act as antagonists in the transport of rhein in Caco-2 cells.

### 3.5. Uptake of Rhein by Caco-2 Cells and Effects of Inhibitors

Results of the cellular uptake of rhein (27.3 *μ*g/ml) into Caco-2 cell monolayers from the A compartment over 2 h are shown in Figure 3(b). After 2 h of incubation, the cellular uptake of rhein was 0.68 ng/*μ*g protein. To investigate the involvement of active efflux in the uptake of rhein, the effects of P-gp, MRP2, and BCRP selective inhibitors on the uptake of rhein were performed. After treatment of Ko143, intracellular accumulation of rhein significantly increased, and no significant changes in the intracellular amounts of rhein were observed after treatment with verapamil or MK-571. With the treatment of Kol43, both the ER value and the intracellular accumulation were significantly altered, suggesting that BCRP may mediate the transport of rhein.

### 3.6. Uptake of Rhein in RE, PE, RPE, and TWCQT by Caco-2 Cells

Further, uptake studies were performed in the presence of RE, PE, RPE, and TWCQT to determine the effects of RE, PE, RPE, and TWCQT on the uptake of rhein (Figure 3(c)). The intracellular accumulation of rhein in RE adding 18.69 *μ*g/ml rhein was approximately equal to that of 27.3 *μ*g/ml rhein alone, suggesting that components in RE did not alter the uptake of rhein in Caco-2 cells. However, the uptake of rhein into Caco-2 cells was significantly reduced in PE, RPE, and TWCQT (*p* < 0.05) compared with rhein at the same dosage, which is consistent with the permeability results. According to these results, we indicate that processed liquorice in TWCQT may inhibit the absorption of rhein in Caco-2 cells to alleviate the toxicity of rhubarb.

## 4. Discussion

Because of the toxicity of rhubarb and related anthraquinones [10], the US Food and Drug Administration (FDA) proposed limits on the dose and duration of usage of dietary supplements containing rhubarb for weight loss. In TCM theories, rhubarb is also considered to possess toxicity, and TWCQT, a multiherb prescription, which consists of rhubarb and other two drugs (processed liquorice and Natrii Sulfas), can be much safer than that of a single-herb rhubarb decoction when it is given at the same dose recorded in TCM [2]. It was pointed out that in the process of the codecoction of prescribed herbs, chemical constituents may change due to the solvent or heating [15]. In certain formulas, liquorice may reduce toxic intermediates when combined with some toxic herbs [16]; however, liquorice may increase the activity of components of some other herbs [17]. In our studies, we found that processed liquorice could increase the dissolution of rhein in RPE and TWCQT decoctions. Rhein is reported to be the major compound absorbed by the body as determined by human plasma analysis after oral administration of the water extract of rhubarb [6] and one of the major poisonous ingredients of rhubarb [18]. The pharmacokinetics of rhein in rats following oral administration of TWCQT showed that the concentration of rhein in plasma was reduced by herbs mixture [11]. These results suggested that some components in the TWCQT might reduce the absorption of rhein in rhubarb. Thus, it is meaningful to determine the potential role of herbs in TWCQT in the intestinal transport of rhein.

Aviello et al. [19] reported that rhein was devoid of cytotoxic and genotoxic effects in human colon adenocarcinoma cells (Caco-2) at 0.1–10 *μ*g/ml; however, at concentrations present in the colon after a human therapeutic dosage of senna, rhein inhibited cell proliferation. In our study, we found that rhein (3.375–200 *μ*g/ml) had no significant cytotoxic effect on proliferating Caco-2 cells with IC50 3.49 ± 0.96 mg/ml.

ATP-binding cassette transporters, including P-gp, MRP, and BCRP abundantly located in the apical membrane of the intestinal epithelium, are crucial to limit toxicant absorption to prevent poisoning. Verapamil and MK-571 are well-known as the inhibitors of P-gp and MRP2, respectively [20]. Ko143, a potent and selective BCRP inhibitor, displays higher than 200-fold selectivity over P-gp and MRP1 transporters [21]. Consistent with the previous study [22], we demonstrated that, in the Caco-2 cells, the permeability of rhein from B to A was significantly higher than that from A to B. In the presence of BCRP (Ko143) or MRP2 inhibitor (MK-571), the permeability of rhein significantly decreased from the B to A direction, suggesting that BCRP and MRP2 may participate in the efflux of rhein. However, MK-571 was reported to inhibit MRPs, P-gp, and BCRP with distinctive potencies. It was also a less effective inhibitor of BCRP [23]. Therefore, the role of MRP2 in the rhein transport requires further study. Our study revealed that the intracellular accumulations of rhein only significantly increased in the presence of Ko143, while they did not increase in the presence of MK-571 or verapamil. These results provided evidence that only BCRP was involved in rhein transport, which were consistent with Ye et al. [22]. Ye et al. found that rhein was more permeable in the B to A side than that in the opposite in the Madin Darby canine kidney (MDCK) II-BCRP cells; however, no significant differences of rhein permeability were observed in two directions in both MDCK II-MDR1 and MDCK II-MRP2 cells [21]. Nevertheless, Van Gorkom et al. reported that rhein was less cytotoxic in the MRP1 overexpressing GLC4/ADR cell line compared to GLC4, MRP1 inhibition with MK571 increased rhein cytotoxicity, and carboxyfluorescein efflux was blocked by rhein. Thus, they concluded that rhein was a substrate for the MRP1 drug efflux pump and was a cytotoxic agent capable of inducing apoptosis [5]. These contradictory results may be explained by different cell lines and assessment methods.

It has been reported that there were no significant differences in *P*_app_ across the Caco-2 cell monolayer between the rhein alone and combinations of rhein, baicalin, and berberine [24]. Meanwhile, rhein has been proven to have no significant effect on the absorption of highly permeable drugs during coadministration, such as ketoprofen, paracetamol, propranolol, verapamil, digoxin, and rhodamine 123. However, furosemide permeability was enhanced by rhein, which may be partly due to the opening of the paracellular spaces and/or effects on active efflux [25]. In our study, we found that processed liquorice could increase the ER and decrease the intracellular accumulations of rhein, implying that the active ingredients from processed liquorice may play an important role in decreasing the absorption of rhein. Liquorice is a widely used herbal medicine native to southern Europe and parts of Asia and has beneficial applications in both the medicinal and the confectionery sectors. Unlike its usage in Europe, liquorice is commonly combined with other herbs in TCM prescriptions, to enhance the effectiveness of other ingredients, to reduce toxicity, and to improve flavor in almost half of Chinese herbal formulas [15]. The “mediation” effect of liquorice has been demonstrated to occur partially through modulation of drug transporter proteins. Liquorice was shown to inhibit P-gp in an* in vivo* ATPase assay [26] and in intestinal mucosa [27]. The inhibitory action of P-gp was enhanced by a combination of liquorice and Kansui [28] or* Daphne genkwa* [29]. However, He et al. reported that liquorice extraction and its main components (glycyrrhizin, glycyrrhetinic acid, and liquiritin) could activate the P-gp and upregulate its expression [30]. Glycyrrhetic acid, the major metabolite of glycyrrhizin, was also found to activate the P-gp [31]. These discrepancies may be related to other chemicals in liquorice or different concentrations or inspection methods. In addition, glycyrrhizin has been demonstrated to increase hepatic glutathione content, possibly by inhibiting MRP2 [32]. As yet, no systematic studies have been published regarding the effects of liquorice and its active components on BCRP. The effect of liquorice and its active components on rhein transport in Caco-2 cells involved with BCRP requires further study. Further studies are also needed to elucidate how processed liquorice exerts its inhibitory action on the transport of rhein in Caco-2 cells.

## 5. Conclusions

In conclusion, our study provided evidence that processed liquorice in TWCQT could decrease the transport of rhein in Caco-2 cells. The active ingredients of processed liquorice may play a critical role in reducing the absorption of rhein to alleviate the toxicity of rhubarb in TWCQT. Because of the involvement of BCRP in rhein transport, we conjectured that some components in processed liquorice could inhibit the transport of rhein, possibly by mediating BCRP. These results would provide new insight into this ancient drug combination in toxicity reduction and clinical use.

## Figures and Tables

**Table 1:** Effects of prepared licorice extract, rhubarb + prepared liquorice extract, and TWCQT on transport of rhein across the Caco-2 cell monolayer (mean ± SD, *n* = 6).

Group	Concentration	*P*_app_ (×10^−6^ cm/s)		ER
		*P*_AB_	*P*_BA_	*P*_BA_/*P*_AB_
Rhein	27.3 *μ*g/ml	2.31 ± 0.13	6.54 ± 0.27	2.83
	54.6 *μ*g/ml	2.58 ± 0.26	7.02 ± 0.58	2.72
	109.2 *μ*g/ml	2.52 ± 0.19	6.72 ± 0.31	2.38

Rhein + PE	27.3 *μ*g/ml + 1 mg/ml	1.28 ± 0.02^*∗*^	4.32 ± 0.15^*∗*^	3.38
	54.6 *μ*g/ml + 2 mg/ml	1.39 ± 0.09^*∗*^	4.92 ± 0.23^*∗∗*^	3.53
	109.2 *μ*g/ml + 4 mg/ml	1.92 ± 0.03^*∗*^	6.14 ± 0.14^*∗*^	3.20

RPE^a^	1 mg/ml	1.48 ± 0.11^*∗*^	4.84 ± 0.14^*∗∗*^	3.27
	2 mg/ml	1.39 ± 0.07^*∗*^	5.09 ± 0.27^*∗*^	3.66
	4 mg/ml	1.73 ± 0.15^*∗*^	5.72 ± 0.14^*∗*^	3.30

TWCQT^b^	1 mg/ml	1.56 ± 0.12^*∗*^	4.94 ± 0.13^*∗∗*^	3.16
	2 mg/ml	1.48 ± 0.09^*∗*^	5.15 ± 0.32^*∗*^	3.48
	4 mg/ml	1.82 ± 0.11^*∗*^	5.82 ± 0.19^*∗*^	3.19

PE: prepared licorice extract, RPE: rhubarb + prepared liquorice extract; TWCQT: Tiaowei Chengqi Tang. ^a^The amounts of rhein in 1, 2, and 4 mg/ml RPE were 27.7, 55.4, and 110.8 *μ*g/ml, respectively. ^b^The amounts of rhein in 1, 2, and 4 mg/ml TWCQT were 27.3, 54.6, and 109.2 *μ*g/ml, respectively; ^*∗*^*p* < 0.05; ^*∗∗*^*p* < 0.01 compared with rhein alone.
